# Molecular Traits of Long Non-protein Coding RNAs from Diverse Plant Species Show Little Evidence of Phylogenetic Relationships

**DOI:** 10.1534/g3.119.400201

**Published:** 2019-06-24

**Authors:** Caitlin M. A. Simopoulos, Elizabeth A. Weretilnyk, G. Brian Golding

**Affiliations:** Department of Biology, McMaster University, Hamilton, Ontario, Canada, L8S 4K1

**Keywords:** lncRNA, CREMA, phylogenetic signal, RNASeq, evolution

## Abstract

Long non-coding RNAs (lncRNAs) represent a diverse class of regulatory loci with roles in development and stress responses throughout all kingdoms of life. LncRNAs, however, remain under-studied in plants compared to animal systems. To address this deficiency, we applied a machine learning prediction tool, Classifying RNA by Ensemble Machine learning Algorithm (CREMA), to analyze RNAseq data from 11 plant species chosen to represent a wide range of evolutionary histories. Transcript sequences of all expressed and/or annotated loci from plants grown in unstressed (control) conditions were assembled and input into CREMA for comparative analyses. On average, 6.4% of the plant transcripts were identified by CREMA as encoding lncRNAs. Gene annotation associated with the transcripts showed that up to 99% of all predicted lncRNAs for *Solanum tuberosum* and *Amborella trichopoda* were missing from their reference annotations whereas the reference annotation for the genetic model plant *Arabidopsis thaliana* contains 96% of all predicted lncRNAs for this species. Thus a reliance on reference annotations for use in lncRNA research in less well-studied plants can be impeded by the near absence of annotations associated with these regulatory transcripts. Moreover, our work using phylogenetic signal analyses suggests that molecular traits of plant lncRNAs display different evolutionary patterns than all other transcripts in plants and have molecular traits that do not follow a classic evolutionary pattern. Specifically, GC content was the only tested trait of lncRNAs with consistently significant and high phylogenetic signal, contrary to high signal in all tested molecular traits for the other transcripts in our tested plant species.

Long non-protein coding RNAs (lncRNAs), a heterogeneous class of regulatory transcripts, remain greatly understudied in plant species. Although these transcripts have been implicated in development and stress responses of plants, only 13 of these transcripts have been empirically functionally characterized to date (Wang and Cheksnova 2017; Nejat and Mantri 2018; Zhao *et al.* 2018). While researchers often focus on computational prediction of these transcripts, particularly lncRNAs expressed under stressful conditions, biological insights on the evolution, mechanisms and function of lncRNAs remain uncertain.

Simopoulos *et al.* (2018) reported that the genome of *Eutrema salsugineum*, an extremophile, contains a lower proportion of putative lncRNAs in comparison to the genome of model plants *Arabidopsis thaliana* and *Oryza sativa*. A lower number of predicted lncRNAs in *E. salsugineum* is surprising due to the naturally high capacity of this species to tolerate extreme environmental conditions (Champigny *et al.* 2013; Kazachkova *et al.* 2018) and the oft-cited association between expressed lncRNAs and stress responses (Wang *et al.* 2017; Xu *et al.* 2017). *E. salsugineum*’s unexpectedly low number of predicted lncRNAs compared to its close and more stress sensitive relative *A. thaliana* leads to questions of potential natural variation in lncRNA number. However, the differences in predictions of lncRNAs in these species may be due to data availability as few plant species have had their reference annotation updated regularly in genomic databases. For example, novel gene information has yet to be updated for *E. salsugineum* since the official reference genome was released in 2013 (Yang *et al.* 2013) although Champigny *et al.* (2013) presented an additional 665 transcriptional units for which the reference genome had no annotation. Recently, Yin *et al.* (2018) have added to the number of novel transcripts in *E. salsugineum* with evidence of expression of an additional 65 transcripts, none of which are available in the reference annotation of *E. salsugineum*.

LncRNAs may be missing from genome annotations because they are difficult to identify due to their low, tissue- and condition-dependent expression (Derrien *et al.* 2012). Further, contrary to protein-coding genes and other non-coding loci, the evolution of lncRNAs is not well understood. Limited nucleotide conservation has been identified in mammalian lncRNAs (Hezroni *et al.* 2015), and structural conservation remains controversial (Rivas *et al.* 2017). Instead of using homology, distinguishing traits such as transcript length (Kapranov *et al.* 2007), open reading frame (ORF) (or lack of) length (Kapranov *et al.* 2007), GC content (Niazi and Valadkhan 2012), and number of exons in a transcript (Derrien *et al.* 2012) are often used in lncRNA prediction studies. Detected phylogenetic signal in traits of transcripts, rather than sequence homology, can indicate that trait values follow the expected evolutionary patterns of tested species. For example, high phylogenetic signal implies traits are more similar in closely related species, whereas low phylogenetic signal suggests the opposite: less similarity in tested traits than expected in closely related species. However, identifying which evolutionary process may be influencing a significant phylogenetic signal is complex and many different processes are associated with both high or low signal estimates (Revell *et al.* 2008).

Phylogenetic signal can be measured using many different indices, however three different approaches are prevalent throughout estimation methods: Brownian motion, an Ornstein-Uhlenbeck process and spatial autocorrelation. High phylogenetic signal detected by a signal estimation method that uses the theory of Brownian motion, or evolution following a random walk, can be observed in both natural selection and genetic drift scenarios (Revell *et al.* 2008). Conversely, low detected phylogenetic signal can be inferred as the lack of similarity in tested traits, as opposed to divergence of traits, and is common in adaptive radiation or other fast adaptive processes (Kamilar and Cooper 2013). First described with applications to evolution by Hansen (1997), the Ornstein-Uhlenbeck process allows for a random walk, similar to Brownian motion, but also for species to evolve toward an adaptive peak or fitness optimum, thus suggesting data that fit an Ornstein-Uhlenbeck process as evidence of an adaptive process. Furthermore, local estimates of Moran’s I, based on the concept of spatial autocorrelation, estimate phylogenetic signal throughout evolutionary time. Positive autocorrelation indicates similarity of trait values at a given phylogenetic distance, while negative autocorrelation suggests dissimilarity at a given phylogenetic distance. Diniz-Filho (2001) has shown, however, that it is the changes in local autocorrelation over phylogenetic distance beyond a significance threshold that are important for evolutionary process inference. A trait following an Ornstein-Uhlenbeck adaptive process would have a reduced phylogenetic distance at which Moran’s I changes in magnitude and crosses a threshold of no significant autocorrelation, also called a “phylogenetic patch”. Thus, it is necessary to test for phylogenetic signal in traits using multiple and diverse estimation methods to infer putative evolutionary processes that may be driving significant signal.

In this study, we predicted lncRNAs from transcriptomes of 11 plant species with widely different evolutionary histories. Transcripts were assembled from RNASeq data without restriction of existing reference annotation in order to obtain a representation of all expressed loci in each study. Transcript sequences were then input into CREMA (Simopoulos *et al.* 2018) for accurate lncRNA prediction and ranking. Unlike other lncRNA prediction tools, CREMA is trained only on experimentally validated lncRNA transcripts and has been tested for use on transcript sequences of multiple plant species. Following lncRNA prediction, we observed that up to 99% of predicted lncRNAs may not be present in their corresponding reference annotation. Thus, we caution that researchers should not rely only on publicly available annotation for lncRNA research. Finally, as there has been little evidence for sequence conservation in lncRNAs between species in different families (Nelson *et al.* 2016), a phylogenetic signal was not expected in the distinguishing molecular traits of lncRNAs, such as transcript length and GC content. However, our comparative study detected a consistently high phylogenetic signal in GC content of lncRNAs with no similarity identified in the other traits tested in these regulatory transcripts. In particular, GC content differences relative to protein-coding RNA represent a trait that could help researchers distinguish putative functional lncRNAs from non-functional and spurious transcription, or fragmented protein-coding RNAs.

## Materials and Methods

### Data collection

RNASeq data from multiple plant species were downloaded from the SRA database (See Table 1 for accession and SRA IDs). All plants in this analysis have a publicly available sequenced genome. All RNASeq reads except for those from *E. salsugineum* were downloaded from the Sequence Read Archive (SRA) (https://www.ncbi.nlm.nih.gov/sra). To be considered for analysis, plants must have been grown under control conditions without being subjected to stress. For consistency, preference was given to studies that used leaf tissue from mature plants, although use of older seedlings was accepted. Leaves or leaf-like tissue represented a shared morphological feature of photosynthetically active cells throughout all tested species and was used throughout the study to ensure an equal opportunity for capturing lncRNA expression. Additionally, only RNASeq reads from Illumina technology were considered, however both paired and single end reads were used.

**Table 1:** Information on the data sources of RNASeq libraries used in this study

Species	# high quality reads	# mapped reads	BioProject	SRA	Source of RNASeq	Genome Source
*Solanum tuberosum*	12,469,853	11,106,056	PRJNA311702	SRR3162008	Sprenger *et al.* (2016)	Sharma *et al.* (2013)
*Solanum lycopersicum*	18,624,814	18,277,415	PRJNA307656	SRR3095793	Cárdenas *et al.* (2016)	Tomato Genome Consortium (2012)
*Eutrema salsugineum*	49,522,792	46,130,371	PRJNA494564	SRR7962298	This manuscript	Yang *et al.* (2013)
*Arabidopsis thaliana*	23,490,825	23,111,430	PRJNA186843	SRR2079778	Woo *et al.* (2016)	Cheng *et al.* (2017)
*Zea mays*	15,141,539	14,481,792	PRJNA269060	SRR1688291	Gonzalez-Munoz *et al.* (2015)	Schnable *et al.* (2009)
*Oryza sativa*	23,501,682	22,145,297	PRJNA301554	SRR2931278	Wilkins *et al.* (2016)	Ouyang *et al.* (2007)
*Amborella trichopoda*	17,913,230	17,355,462	PRJNA212863	SRR5293262	Amborella Genome Project (2013)	Amborella Genome Project (2013)
*Selaginella moellendorffi*	108,008,790	92,873,912	PRJNA351923	SRR4762345	James *et al.* (2017)	Banks *et al.* (2011)
*Physcomitrella patens*	10,520,395	8,243,406	PRJNA265205	SRR1553300	Frank and Scanlon (2015)	Lang *et al.* (2018)
*Chlamydomonas reinhardtii*	22,002,690	21,222,625	PRJNA264777	SRR1622084	Panchy *et al.* (2014)	Merchant *et al.* (2007)
*Boea hygrometrica*	16,972,867	15,594,598	PRJNA210992	SRR929426	Xiao *et al.* (2015)	Xiao *et al.* (2015)

*E. salsugineum* reads were sequenced using Illumina technology from Shandong ecotype rosette leaves grown under control, unstressed conditions as outlined in MacLeod *et al.* (2015). Fully-expanded leaves were used for RNA sequencing, and were collected between 8 and 10 hr into the day cycle. RNA was extracted from leaves flash-frozen using liquid nitrogen using a modified hot borate method as described by Champigny *et al.* (2013). A quality control analysis was completed on the RNA using RNA Nano 600 chips on a Bioanalyzer 2100 and purified using three on-column purifications by Genelute mRNA miniprep kit (Cat. No. MRN10, Sigma). Finally, preparation of cDNA for sequencing was performed with the NEBNext multiplex cDNA synthesis kit for Illumina using random hexamers (Cat. No. E7335, New England Biolabs, Ipswich, MA). Cleanup of fragmented RNA was performed with Agencourt AMPure XP Beads (Cat. No. 163987, Beckman Coulter, Mississauga, ON) following the manufacturer’s protocol. Raw FASTQ files were deposited to the SRA with submission ID SRR7962298 and BioProject accession PRJNA494564.

### Transcript assembly and lncRNA prediction

Reads from all plant species were trimmed using Trimmomatic v0.36 (Bolger *et al.* 2014) and aligned to their corresponding genomes using STAR v2.5.2b (Dobin *et al.* 2013) with default settings other than–outFilterIntronMotifs set to RemoveNoncanonical and–alignEndsType EndtoEnd. Aligned reads were assembled into transcripts by StringTie v1.3.4d (Pertea *et al.* 2015). GTF files of assembled transcripts were merged with GTF files of annotated genomes and are stored on GitHub: https://github.com/caitsimop/lncRNA-compGenomics. Alignment quality was tested using gffcompare v0.10.4 (https://github.com/gpertea/gffcompare) by comparing assembled transcript GFF files with reference genome GFF files. Alignment quality metrics were used to confirm alignment quality and transcript assembly quality using accuracy and precision values (File S2). Gffcompare output was also used to identify novel transcripts and to quantify transcript exon numbers in each RNASeq library.

### Identifying lncRNAs From RNASeq data

Assembled transcript sequences were input into CREMA (https://github.com/gbgolding/crema) (Simopoulos *et al.* 2018) for ranked lncRNA prediction. The number of lncRNAs in each species was calculated as a percentage of all transcripts (the sum of novel assembled transcripts and transcripts in reference annotation). The percentage of lncRNAs was used for normalization across all studied plant species to ensure appropriate comparisons to species with different sized transcriptomes.

### Phylogenetic signal in lncRNA traits

Four continuous molecular traits were chosen for phylogenetic signal analysis on predicted lncRNAs: 1. Number of exons in transcript, 2. GC content of transcript, 3. Length of transcript, and 4. Length of maximal ORF. Features were extracted from transcript sequences and gffcompare outputs using a custom Python script. The phylosignal R package (Keck *et al.* 2016) was used to detect phylogenetic signal in lncRNAs, all other transcripts, and the differences between lncRNAs and all other transcripts for each trait in all species except for *Boea hygrometrica*. Separate phylogenetic signal tests were completed for each trait. Although we expect there to be correlation between transcript length and ORF length, we did not observe a correlation, particularly in lncRNAs. Phylogenetic signal of the mean value of the four traits was calculated using three separate methods: Moran’s I (Moran 1948; Gittleman and Kot 1990), Blomberg’s K (Blomberg *et al.* 2003) and Pagel’s λ (Pagel 1999). Local autocorrelation estimates at 100 phylogenetic distance points were also computed using Moran’s I and phylosignal to identify the location and sign of the detected autocorrelation. To identify significant autocorrelation estimates, 1000 bootstrap replicates were used for 95% confidence interval calculation. Autocorrelation estimates were considered significant if 95% confidence intervals did not overlap the null hypothesis threshold of -0.111. The null hypothesis that there is no detectable phylogenetic signal, or, autocorrelation, was a threshold of −1/(n−1) where n=10, or the number tested species, as suggested by Keck *et al.* (2016). Because branch lengths were required by the phylosignal package, branch lengths were estimated using the dnaml program in PHYLIP (Felsenstein 1993) and a MAFFT v.7.205 (Katoh *et al.* 2002) alignment of *rps16*, *atp2*, *18s*, *26s* and *SMC1* (File S4). *B. hygrometrica* was not included in phylogenetic signal analysis due to the limited percentage of annotated loci in genome annotation. The tree topology of land plants as reported by the Amborella Genome Project (2013) was used, and branch lengths were estimated from this topology. Branch lengths representing site changes were converted to relative age of branches using the R package ape (Paradis and Schliep 2019). A lambda value of 0 was chosen from 0, 0.1 and 1 after testing for the lowest log likelihood of lambda options.

Trait values with high K values (K >1) were chosen for further testing for better fit to models that consider an Ornstein-Uhlenbeck process and may indicate selection on traits. Traits values were fit with macroevolutionary models using the geiger (Harmon *et al.* 2008) R package and the fitContinuous function. Both “BM” (Brownian motion) and “OU” (Ornstein-Uhlenbeck) models were considered. Log likelihood estimates were used to test for the goodness of fit for “BM” and “OU” models while also considering the number of parameters that each model contains.

### Data Availability

Raw FASTQ RNA sequencing data are available in the SRA with submission ID SRR7962298 and BioProject accession PRJNA494564. Ranked lncRNA prediction scores and GFF files of assembled transcripts are available on the author’s GitHub https://github.com/caitsimop/lncRNA-compGenomics. Classifications of lncRNAs made by gffcompare are made available in File S1. Quality of transcriptome assemblies are available in File S2. The FASTA file containing sequences of the genes used in the estimation of branch lengths is available in File S3. The phylogenetic tree with branch lengths adjusted relative to time is found in Figure S4. Supplemental material available at FigShare: https://doi.org/10.25387/g3.8250953.

## Results

### Multispecies lncRNA prediction

**Figure 1:**
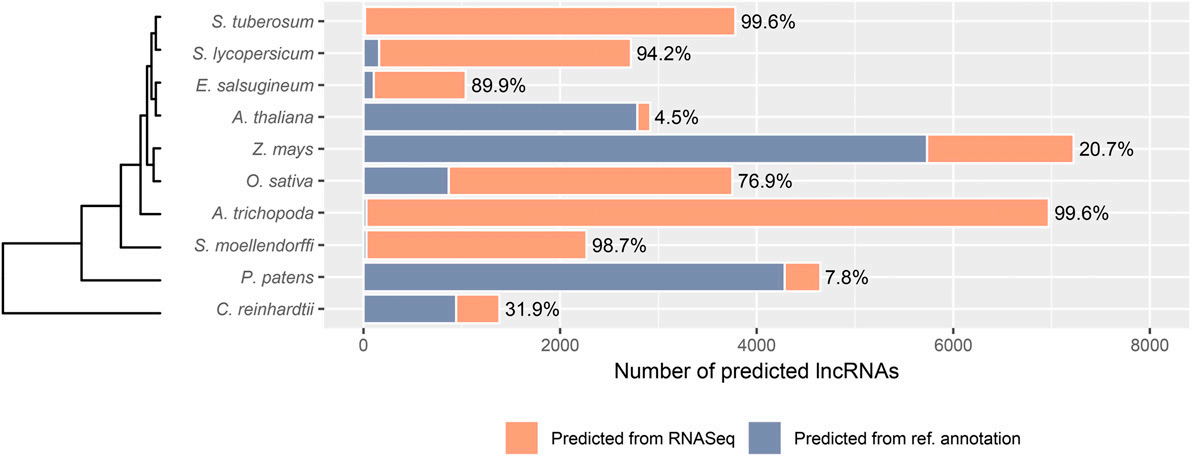
Total predicted lncRNAs from 10 plant species. The counts of putative lncRNAs are categorized by transcripts that appear in the reference annotation of each species (purple) and novel transcripts, or those that did not appear in transcriptome annotation (coral). The percentages of novel transcripts (coral) predicted as lncRNAs appear above each bar.

We chose plant species with diverse and divergent evolutionary histories for lncRNA prediction comparisons. Specifically, we included Angiosperms (both monocots and dicots), a Lycophyte, a Bryophyte and an algal species for this work (see Table 1 in Materials and Methods). As novel transcripts were of importance to this work, RNASeq data from published experiments were used when available to assemble transcripts or were prepared *de novo* as in the case of one species (*E. salsugineum*). To be chosen for this study, data from the SRA database had to include samples produced with Illumina sequencing technology. Species chosen must have publicly available reference genome sequences and corresponding annotation. For consistency, leaf tissue from mature plants was preferred but younger leaves, or entire organisms in the case of *Chlamydomonas reinhardtii*, were used. To remove any potential biases toward transcripts induced by stress, only control, unstressed samples were chosen for analyses. After read mapping to appropriate plant genomes, transcripts were assembled using StringTie allowing for identification of novel transcripts. Sequences of assembled transcripts were input into CREMA, a lncRNA prediction tool (Simopoulos *et al.* 2018) and total lncRNA numbers in each plant species are described in Figure 1 and Table 2. Ranked prediction scores of all transcripts in each species are available on GitHub: https://github.com/caitsimop/lncRNA-compGenomics. The percentage of total transcripts predicted as lncRNAs range from 3% in *E. salsugineum* to 16.6% in *Amborella trichopoda* with a mean percentage of 6.4% ±1.1% for the 11 analyzed plant species (Table 2). On average, 52% of the predicted lncRNAs were found in intergenic regions leaving almost half of the predicted lncRNAs overlapping with a protein coding gene or as putative splicing variants (File S1).

**Table 2:** Number of predicted lncRNAs in each species ordered by phylogenetic relationship

Species	# of assembled transcripts	# predicted lncRNAs	% lncRNAs
*Solanum tuberosum*	73,656	3,783	5.1%
*Solanum lycopersicum*	43,936	2,721	6.2%
*Eutrema salsugineum*	34,862	1,040	3.0%
*Arabidopsis thaliana*	61,480	2,918	4.8%
*Zea mays*	95,713	7,225	7.6%
*Oryza sativa*	66,562	3,753	5.6%
*Amborella trichopoda*	42,118	6,972	16.6%
*Selaginella moellendorffi*	33,266	2,269	6.8%
*Physcomitrella patens*	88,649	4,648	5.2%
*Chlamydomonas reinhardtii*	21,467	1,383	6.4%
*Boea hygrometrica**^a^*	58,531	1,796	3.0%

a We did not complete phylogenetic analysis on *B. hygrometrica* due to limited gene annotation availability, and thus it is placed at the end of the table.

To determine how reference annotation may affect lncRNA research in plant systems, all assembled transcripts from each species, including novel transcripts, were compared to those found in the corresponding reference annotation. Transcripts that were not found in reference annotation and also predicted as a putative lncRNA were identified and are referred to as “novel” lncRNAs throughout this manuscript. The proportion of novel lncRNA in all predicted lncRNAs ranged among species from a low 4.5% predicted in *A. thaliana* and a high 99.6% in *Solanum tuberosum* (Figure 1). Because *A. thaliana* is a well studied model plant with an almost fully annotated genome, we expected this species to have fewer novel transcripts assembled from the RNASeq data. Additionally, we expected that most lncRNAs predicted from the *A. thaliana* transcriptome to already be found in the reference annotation. The high percentage of lncRNAs found in the reference annotation of *A. thaliana* indicates that CREMA makes accurate predictions and suggests that the lower percentages of known lncRNAs identified in the other species are due to incomplete annotations (Figure 1).

### Phylogenetic signal in molecular traits of plant lncRNAs

**Figure 2:**
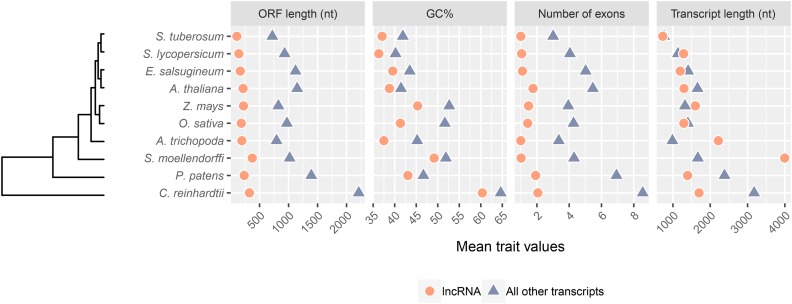
Mean trait values of transcripts predicted as lncRNAs (coral, circle) and all other assembled transcripts (purple, triangle). Species are ordered as per phylogenetic relationships.

We first considered overall trends in some typical distinguishing traits of lncRNAs: ORF length, GC content, number of exons, and transcript length. All species showed a similar trend where putative lncRNAs had a lower GC%, fewer exons, and shorter ORF length compared to the other transcripts in their corresponding transcriptomes (Figure 2). Length of transcripts, however, deviated from this trend where *Zea mays*, *Selaginella moellendorffi* and *A. trichopoda* all have putative lncRNAs longer than other transcripts in their transcriptome (Figure 2). Deviation from the expected trend of shorter lncRNA transcripts in three of the selected species suggests that transcript length may not be a useful distinguishing trait in lncRNA prediction.

We tested for phylogenetic signal in mean trait values of the four molecular traits previously mentioned in both lncRNAs and all transcripts other than lncRNAs. Phylogenetic signal estimates were calculated using three different methods that employ two different models of evolution, Moran’s I, Pagel’s λ and Blomberg’s K. We estimated phylogenetic signal in all species but *B. hygrometrica* due to the incomplete status of its genome annotation.

Since each phylogenetic signal estimation method is based on different concepts, all estimates cannot be interpreted the same. First, Moran’s I is a measure of autocorrelation (Gittleman and Kot 1990). Autocorrelation, when referring to phylogenetic signal, indicates how correlated traits are in terms of phylogenetic distance. Due to the use of 10 species, Moran’s I must be compared to a calculated threshold of -0.111 (Keck *et al.* 2016) to determine significant autocorrelation and phylogenetic signal. A significant estimate greater than -0.111 indicates positive significant global autocorrelation and that trait values of closely related species are more similar to each other. Conversely, a significant estimate less than -0.111 suggests global significant negative autocorrelation. The Brownian motion model, originally used to describe the motion of particles suspended in fluid, is another model used to describe how traits evolve through time. In the case of phylogenetic signal, a trait following the Brownian motion model exhibits a random walk where the value of the trait can change in any direction at any time. Pagel’s λ uses this Brownian motion model and can be interpreted as the transformation the phylogeny requires to explain trait distribution if the trait followed Brownian motion (Pagel 1999). Thus, a value of 1 would indicate a phylogeny as expected under Brownian motion and high phylogenetic signal, and a significant value of 0 would mean a trait distribution that does not follow Brownian motion, consequently, low phylogenetic signal. Finally, Blomberg’s K, which also uses the Brownian motion model, can be interpreted as the ratio of observed values over expected values if the trait follows the Brownian motion model (Blomberg *et al.* 2003). A value of K = 1 can be interpreted as a trait distribution following Brownian motion, and as K becomes larger than 1, a stronger signal is detected. Conversely a value of K < 1 indicates low phylogenetic signal, and less similarity between closely related tested species.

Table 3 shows phylogenetic signal estimates using all three methods for each of the four molecular traits. The mean trait values and phylogenetic relationships of the tested species are presented in Figure 2. In predicted lncRNAs, GC content was the only trait that demonstrates high phylogenetic signal using all phylogenetic signal detection methods (Table 3). While ORF length was had a significant positive global autocorrelation (I = 0.040; Table 3), a value of K < 1 indicates less similarity than expected under Brownian motion, suggesting unclear phylogenetic signal estimation. Blomberg’s K also suggests less similarity than expected in the number of exons of lncRNAs, however no other method indicated detectable significant phylogenetic signal. Transcript lengths of lncRNAs in tested species also demonstrate a moderate positive global autocorrelation. Conversely, all four traits consistently had significant phylogenetic signal estimates when all transcripts other than lncRNAs were evaluated, although λ for ORF length, number of exons and transcript length were slightly less than 1.

**Table 3:** Phylogenetic signal estimates in transcript features

Feature*^a^*	lncRNA			All other transcripts		
	I	λ	K	I	λ	K
ORF	0.040*	0.975	0.621*	0.010*	0.974*	1.746*
GC%	0.032*	1.027*	1.614*	0.048*	1.020*	1.038*
Exons	−0.053	0.620	0.336*	0.010*	0.922*	1.068*
Length	−0.020*	1.007	0.642	0.038*	0.953*	1.436*

* *P* < 0.05.

I = Moran’s I, K = Blomberg’s K, λ = Pagel’s λ

a Where “ORF“ refers to ORF length,“ GC%“ refers to GC content, “Exons“ refers to number of exons and “Length” refers to transcript length.

### Evolutionary processes and phylogenetic signal

We examined traits with an estimated K > 1 with an evolutionary model that may suggest natural selection, the Ornstein-Uhlenbeck process (Hansen 1997), because high phylogenetic signal defined as K > 1 (Kamilar and Cooper 2013) can indicate similarity by both genetic drift and natural selection. In lncRNAs, GC content is the only trait with K > 1, and has significant phylogenetic signal detected using all three methods (Table 3). We compared the fit of a Brownian motion model *vs.* an Ornstein-Uhlenbeck model in our data using log likelihood values and a chi square test for significance estimates. Although the Ornstein-Uhlenbeck model had the smallest log-likelihood, a chi square test indicated that there was no significant fit difference when comparing the Brownian motion and Ornstein-Uhlenbeck model (*P* = 0.81). Because the Brownian motion model has the least number of parameters, this suggests that a Brownian motion model is the most reasonable fit for the data, and there is a lack of evidence for an adaptive process.

All four traits of all transcripts other than lncRNAs had significant high phylogenetic signal when estimated using Blomberg’s K (K >1), therefore we also tested for a better fit explained by the Ornstein-Uhlenbeck process. Again, the Ornstein-Uhlenbeck process was not a significantly better fit than a Brownian motion model when considering log likelihood values and a chi-square test (ORF length: *P* = 1, GC content: *P* = 1, number of exons: *P* = 1, transcript length: *P* = 0.75).

**Figure 3:**
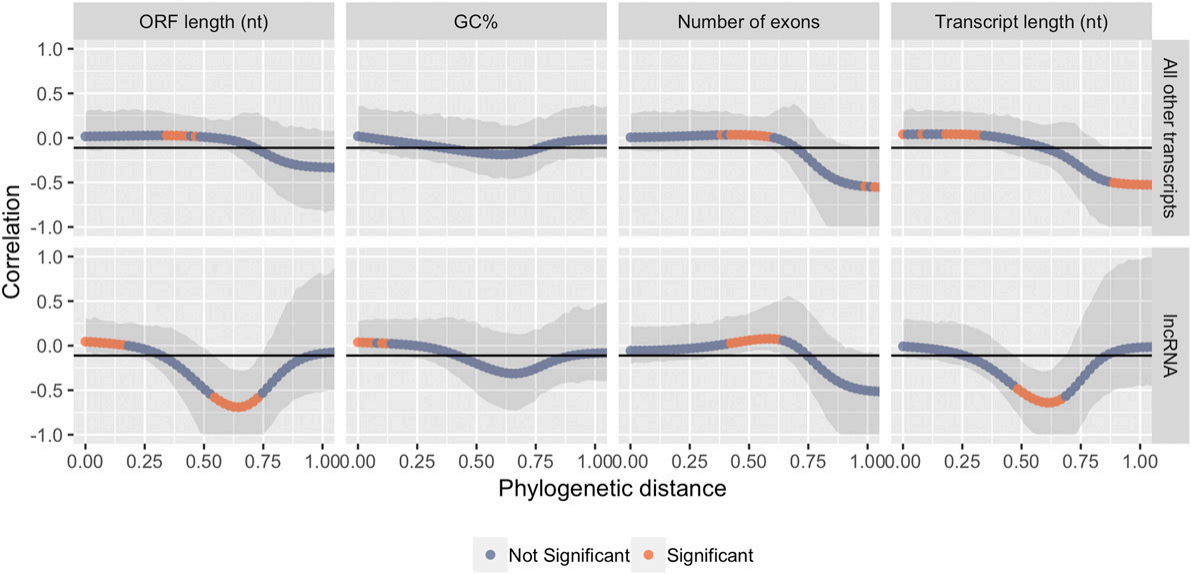
Moran’s I local correlogram of mean trait values in lncRNAs and All Other Transcripts. Coral points indicate significant phylogenetic signal at a particular phylogenetic distance. The horizontal line represents a value of the null hypothesis that no phylogenetic signal is detected. The null hypothesis value is -0.111, or −1/(n−1) where n=10, or the number of tested species. The 95% confidence intervals, computed using bootstrapping, are also plotted and were used to identify significant values.

We tested for local autocorrelation at 100 phylogenetic distances considering the most recent common ancestors of all species as a robust approach to an analysis on a small phylogeny. Confidence intervals were computed using 1000 bootstrapping replicates for a non-parametric significance estimate using a calculated threshold of -0.111. Figure 3 visualizes the local correlations of traits in both lncRNAs and all other transcripts and are limited to the phylogenetic distances of the tested phylogeny (0-1 phylogenetic distance). We detected significant positive local autocorrelation at short phylogenetic distances in ORF length and GC content of lncRNAs (Figure 3). This suggests that closely related species contain lncRNAs with similar ORF lengths and GC content. There was no consistent significant autocorrelation at any short phylogenetic distances in any tested traits in all other transcripts (Figure 3). Detected phylogenetic patches are shorter in the ORF and transcript lengths of lncRNAs compared to all other transripts. The opposite is true in the GC content of lncRNAs, where longer phylogenetic patches are observed. Shorter phylogenetic patches suggest an adaptive process as described by an Ornstein-Uhlenbeck model, rather than genetic drift.

## Discussion

We used raw RNASeq data from multiple independent studies to make inferences on the numbers of predicted lncRNAs in 11 phylogenetically divergent plant species, and to identify putative phylogenetic signal in these regulatory loci. Our data-mining approach enabled us to use the same protocols for read mapping, transcript assembly, and lncRNA prediction for each species. In performing the same read-mapping and lncRNA prediction protocols, we were able to address a concern raised by Kapusta and Feschotte (2014) that comparisons between lncRNA numbers in animals can be misleading when prediction numbers are products of meta-analyses involving different prediction methods and lncRNA criteria. We found that the percentage of transcripts predicted as lncRNAs was, on average, 6.4% with two outliers: *A. trichopoda* with 16.6% and *E. salsugineum* with 3%. These estimates for lncRNA contributions to plant genomes are lower than comparable predicted values for humans, where Gencode (Harrow *et al.* 2012) v29 (https://www.gencodegenes.org) and NONCODE v5 (Fang *et al.* 2017) identified 29,566 and 172,216 putative lncRNA transcripts, respectively. However, there is a lack of evidence for function in these thousands of transcripts as they were identified by a transcript filtering method and the majority remain without experimental validation. In particular, NONCODE v5 has predicted function in only 1961 of their identified lncRNAs. Nonetheless, due to the methods used regarding read mapping, our results rely on the genome quality of each species. Fragmented genomes, as seen in *A. trichopoda*, *E. salsugineum* and *S. moellendorffi*, may have limited our predictive abilities, and low predicted lncRNA numbers may in part be due to data availability.

The review by Kapusta and Feschotte (2014) also included a meta-analysis describing variation in predicted lncRNA numbers among multiple animal species, a comparison similar to our observed prediction numbers in plants. In addition to their concern about transcriptome data arising from different methodologies, Kapusta and Feschotte (2014) also raised the issue of temporal and location specific lncRNA expression. We share a comparable concern in that plant lncRNAs have yet to be predicted in all tissue types for all developmental time points in all possible environments, so undoubtedly the number of putative lncRNAs detected in plants will increase over time. For example, in our study, we identified 2918 putative lncRNAs in *A. thaliana* plants that were grown under conditions designed to avoid exposing plants to sources of stress. In contrast, although using different prediction methods, Zhao *et al.* (2018) identified 6150 putative lncRNAs in *A. thaliana* plants undergoing cold, ABA and drought treatments. This difference in predicted lncRNAs is consistent with the expectation that lncRNAs likely play a role in stress responses and hence one expects to find increased diversity and abundance of lncRNAs in stressed relative to unstressed plants. Interestingly, Zhao *et al.* (2018) found that lncRNAs in *A. thaliana* are shorter and have fewer exons than all other transcripts, observations that agree with our study (Figure 2). Thus our machine learning-based methodology that was trained on only empirically characterized, functional lncRNAs and the filtering method employed by Zhao *et al.* (2018) led to similar conclusions on traits shared by lncRNAs that distinguish them from other transcripts. This finding is also consistent with our previous, cross-validation estimates of CREMA’s accuracy (96%) and specificity (0.994) (Simopoulos *et al.* 2018) making CREMA a suitable method for lncRNA prediction from RNASeq data.

The genome of *A. trichopoda*, the sister taxon to all other extant angiosperms, represents a species with a unique evolutionary history. During genome annotation, The Amborella Genome Project (2013) observed a larger number of the atypical 23 to 24nt plant miRNAs than expected as they were found in two times greater frequency than any other land plant. Additionally, eight predicted miRNA families in *A. trichopoda* have evidence of loss in more recent angiosperms (Amborella Genome Project 2013). The excess of miRNAs in *A. trichopoda* may reflect the high proportion of lncRNAs predicted in this study (at 16%; Table 2) as miRNA progenitors are considered to be lncRNAs (Saini *et al.* 2008).

Two plants, namely *E. salsugineum* and *B. hygrometrica*, were found to have the lowest proportion of lncRNAs in their transcriptomes (Table 2). *E. salsugineum* represents a plant with a halophytic life strategy and a capacity to tolerate a variety of extreme environmental conditions (Kazachkova *et al.* 2018). Indeed, *E. salsugineum* has been used as a model plant in stress response studies due to its naturally high tolerance to abiotic stresses such as salt (Taji *et al.* 2004), cold (Griffith *et al.* 2007), drought (MacLeod *et al.* 2015), and nutritional deficiencies (Velasco *et al.* 2016). Moreover, *E. salsugineum* shows constitutive expression of genes reported to be stress-responsive in many plants (Taji *et al.* 2004; Gong *et al.* 2005; Wong *et al.* 2006; Velasco *et al.* 2016). *B. hygrometrica*, aptly named “the resurrection plant”, is also considered an extremophile by virtue of its capacity to survive desiccation (Xiao *et al.* 2015). However, *B. hygrometrica* shows different expression pattern changes when experiencing stress compared to *E. salsugineum*. Zhu *et al.* (2015) did not observe constitutively high expression of stress tolerance genes in *B. hygrometrica* during desiccation. Instead, *B. hygrometrica* seemed to require gradual dehydration priming for survival after rehydration post-desiccation (Zhu *et al.* 2015). *B. hygrometrica* plants that have been subsequently rehydrated after this dehydration “training” have expression patterns more similar to desiccated plants than those without drought priming (Zhu *et al.* 2015). In other words, after experiencing a first gradual dehydration there are expression differences between well-watered *B. hygrometrica* plants and ones that experienced desiccation. The observation that *B. hygrometrica* plants can show “preparedness” among expressed genes normally responsive to a stressful condition is somewhat analogous to the constitutive nature of expressed genes in *E. salsugineum*. Specifically, *E. salsugineum* plants grown in the absence of high salt display the expression of genes reported to be salt-responsive in other plants (Taji *et al.* 2004; Wong *et al.* 2006). Interestingly, both *E. salsugineum* and *B. hygrometrica* display a low proportion of predicted lncRNAs in their unstressed transcriptomes suggesting a possible connection between high natural stress tolerance and low lncRNA number. Conceivably, with stress-related genes constantly expressed under a primed condition, a plant adapted to an extreme environment may not require the precise regulation conferred by the recruitment of diverse lncRNAs, an important role proposed for the function of lncRNAs in plant stress responses. This hypothesis merits further work as little is known of the importance, or lack thereof, of lncRNAs in extremophile species.

*E. salsugineum* and *A. trichopoda* have distinct evolutionary patterns and yet both species have few putative lncRNAs present in their reference annotations. In total, five of the ten tested plant species had less than 50% of predicted lncRNAs in their respective genome annotations. As genome annotation often relies on homology of predicted genes for functional annotation (Bolger *et al.* 2018), particularly homology to *A. thaliana* protein-coding genes, lncRNAs can often be left out of genome annotation. Researchers studying plant lncRNAs frequently rely on bioinformatic analyses to assemble novel transcripts for lncRNA prediction (Liu *et al.* 2018; Shuai *et al.* 2014), indicating that missing lncRNA annotation should be taken into consideration in forthcoming genome annotation projects. Similar to our work, Jackson *et al.* (2018) recently described misannotation of lncRNAs in mammalian genomes. Gaps in annotation and the ensuing problem with lncRNA identification is exacerbated by the fact that lncRNAs do not follow classic evolutionary conservation. Instead, in both plant and animal systems, lncRNAs often depict a positional conservation pattern, even with minimal sequence conservation, making functional predictions also difficult (Hezroni *et al.* 2015; Mohammadin *et al.* 2015). A lack of extensive lncRNA conservation between species led to our investigation into phylogenetic signal detection in molecular traits of lncRNAs.

In plant species, lncRNA homology has been shown to be virtually non-existent outside of the family classification. Only 1% of predicted *A. thaliana* (Brassicaceae) lncRNAs were identified as homologous in *Tarenaya hassleriana* (Cleomaceae) (Nelson *et al.* 2016). Interestingly, although distantly related, human and plant lncRNAs bear similarities in number of exons, and transcript and ORF length. For example, Derrien *et al.* (2012) describe human lncRNAs as being shorter than protein coding genes, and by commonly having fewer exons which was also observed in our plant species analyses (Figure 2). However, human lncRNAs are typically spliced and most often have two exons, while our study suggests plant lncRNAs are more often unspliced and comprised of a single exon (Figure 2). Domination of single exon novel lncRNAs in our work may be indication of genomic contamination in source data or bioinformatic transcript assembly artifacts. Nevertheless, genomic contamination is unlikely seen in all ten independent experiments, and transcript assembly artifacts more likely result from *de novo* assembly rather than genome guided mapping. Further, Haerty and Ponting (2015) also observed a lower GC content in lncRNAs than the protein coding genes of metazoan lncRNAs, with single-exon lncRNAs having the lowest GC content of all exon-types, reminiscent of the uni-exonic plant lncRNA majority of our analyses. However, the extent to which conserved traits typify plant and animal lncRNAs is difficult to assess at present. CREMA is trained on only validated lncRNA and may be subject to a prediction bias to animal-like lncRNA sequences given that non-plant sources currently comprise the majority of validated lncRNAs (Simopoulos *et al.* 2018). As more plant lncRNA undergo validation, the extent of conservation among lncRNAs from diverse organisms will be easier to detect and estimate with greater precision.

Despite these concerns over bias, among the lncRNAs predicted by CREMA we found significant phylogenetic signal by at least one method in all four tested traits of lncRNAs: ORF length, GC content, the number of exons and transcript length (Table 3). High phylogenetic signal detection in traits of lncRNAs was not expected given the lack of evidence for sequence conservation in this class of RNA (Nelson *et al.* 2016; Hezroni *et al.* 2015). Importantly, because the phylogenetic relationships of species are influencing the tested traits as indicated by detectable phylogenetic signal, the species can no longer be considered independent and statistical methods that assume independence cannot be used. While it is possible to detect phylogenetic signal in our data, it is difficult to infer evolutionary processes from phylogenetic signal estimates as many unique processes can invoke a similar signal estimation (Revell *et al.* 2008). However, high phylogenetic signal and consequent high similarity of GC content in lncRNAs of closely related species is not unexpected, as Haerty and Ponting (2015) demonstrated evidence of selection on the GC content of intergenic lncRNAs in animal species. Additionally, as previously mentioned, the GC content of animal lncRNAs is lower than that of protein-coding genes, mirroring what our work identified in plant lncRNAs.

A lack of similarity in ORF length, number of exons, and transcript length of lncRNAs in close relatives may be due to a variety of processes, including but not limited to: stabilizing selection with high selective strength, selection with variable strength that is bounded by phenotypic limits, punctuated divergent selection, or genetic drift of which rate of drift began low and increased toward present time (Revell *et al.* 2008). Because of the variety of possible complex interpretations of phylogenetic signal and process, Revell *et al.* (2008) do not recommend over-interpretting evolutionary processes from signal data. We have found, however, unique patterns in the phylogenetic signals of molecular traits of lncRNAs compared to all other transcripts in plant species that imply lncRNAs are not following similar evolutionary trends as most other transcripts. Moreover, the lack of similarity in three of the four tested molecular traits in lncRNAs is of interest and this observation implies that evolution of lncRNAs could be species specific, and is not easily defined by an over-arching evolutionary process. On the other hand, it is possible that there are subclasses of lncRNAs with conserved molecular traits yet to be defined due to a lack of validated transcripts.

Because CREMA (Simopoulos *et al.* 2018) predicts lncRNAs using a complex ensemble machine learning model that initially uses ORF length, GC content and transcript length as features for transcript classification, it is possible that high detected phylogenetic signal in these features is a product of the lncRNA prediction tool. However, CREMA’s logistic regression ensemble classifier that is used for the final lncRNA prediction does not use molecular traits as prediction features, but instead binary outputs from eight gradient boosting models. Additionally, we have identified low phylogenetic signal in two of these three molecular traits (Table 3) suggesting that CREMA is able to predict lncRNAs with varying ORF and transcript lengths.

In this work we show that the annotation status of plant species can affect lncRNAs prediction with up to 99% of predicted lncRNAs missing from reference annotation. While researchers may be striving to increase the volume of lncRNA research, the effort to annotate genomes with lncRNAs is not reflective of the increased interest in this RNA class. As such, we caution researchers interested in these regulatory loci to be wary of relying solely upon genome and transcriptome annotations for lncRNA identification. Additionally, our work shows that plant lncRNAs have inconsistent detectable phylogenetic signal in sequence traits, further confirming the complex evolutionary history of lncRNAs. In particular, the differences in detected phylogenetic signal in lncRNAs compared to all other transcripts suggests that lncRNAs evolve, on average, differently than other loci.

## References

[bib1] Amborella Genome Project, 2013 The *Amborella* genome and the evolution of flowering plants. Science 342: 1241089 10.1126/science.1241089 24357323

[bib2] Banks J., Nishiyama T., Hasebe M., Bowman J., Gribskov M., 2011 The *Selaginella* genome identifies genetic changes associated with the evolution of vascular plants. Science 332: 960–963. 10.1126/science.1203810 21551031 PMC3166216

[bib3] Blomberg S., Garland T. Jr., and Ives A. R., 2003 Testing for phylogenetic signal in comparative data: behavioral traits are more labile. Evolution 57: 717–745. 10.1111/j.0014-3820.2003.tb00285.x 12778543

[bib4] Bolger A., Lohse M., and Usadel B., 2014 Trimmomatic: a flexible trimmer for Illumina sequence data. Bioinformatics 30: 2114–2120. 10.1093/bioinformatics/btu170 24695404 PMC4103590

[bib5] Bolger M., Arsova B., and Usadel B., 2018 Plant genome and transcriptome annotations: from misconceptions to simple solutions. Brief. Bioinform. 19: 437–449. 28062412 10.1093/bib/bbw135 PMC5952960

[bib6] Cárdenas P., Sonawane P., Pollier J., VandenBossche R., Dewangan V., 2016 GAME9 regulates the biosynthesis of steroidal alkaloids and upstream isoprenoids in the plant mevalonate pathway. Nat. Commun. 7: 10654 10.1038/ncomms10654 26876023 PMC4756317

[bib7] Champigny M., Sung W., Catana V., Salwan R., Summers P., 2013 RNA-Seq effectively monitors gene expression in *Eutrema salsugineum* plants growing in an extreme natural habitat and in controlled growth cabinet conditions. BMC Genomics 14: 578 10.1186/1471-2164-14-578 23984645 PMC3846481

[bib8] Cheng C., Krishnakumar V., Chan A., Thibaud-Nissen F., Schobel S., 2017 Araport11: a complete reannotation of the *Arabidopsis thaliana* reference genome. Plant J. 89: 789–804. 10.1111/tpj.13415 27862469

[bib9] Derrien T., Johnson R., Bussotti G., Tanzer A., Djebali S., 2012 The GENCODE v7 catalog of human long noncoding RNAs: analysis of their gene structure, evolution, and expression. Genome Res. 22: 1775–1789. 10.1101/gr.132159.111 22955988 PMC3431493

[bib10] Diniz-Filho J., 2001 Phylogenetic autocorrelation under distinct evolutionary processes. Evolution 55: 1104–1109. 10.1111/j.0014-3820.2001.tb00630.x 11475046

[bib11] Dobin A., Davis C., Schlesinger F., Drenkow J., Zaleski C., 2013 STAR: ultrafast universal RNA-seq aligner. Bioinformatics 29: 15–21. 10.1093/bioinformatics/bts635 23104886 PMC3530905

[bib12] Fang S., Zhang L., Guo J., Niu Y., Wu Y., 2017 NONCODEV5: a comprehensive annotation database for long non-coding RNAs. Nucleic Acids Res. 46: D308–D314. 10.1093/nar/gkx1107 PMC5753287 29140524

[bib13] Felsenstein J., 1993 *PHYLIP (phylogeny inference package)*, *version 3.5 c*, Joseph Felsenstein. University of Washington.

[bib14] Frank M., and Scanlon M., 2015 Transcriptomic evidence for the evolution of shoot meristem function in sporophyte-dominant land plants through concerted selection of ancestral gametophytic and sporophytic genetic programs. Mol. Biol. Evol. 32: 355–367. 10.1093/molbev/msu303 25371433

[bib15] Gittleman J. L., and Kot M., 1990 Adaptation: statistics and a null model for estimating phylogenetic effects. Syst. Zool. 39: 227–241. 10.2307/2992183

[bib16] Gong Q., Li P., Ma S., Indu Rupassara S., and Bohnert H. J., 2005 Salinity stress adaptation competence in the extremophile *Thellungiella halophila* in comparison with its relative *Arabidopsis thaliana*. Plant J. 44: 826–839. 10.1111/j.1365-313X.2005.02587.x 16297073

[bib17] Gonzalez-Munoz E., Avendano-Vazquez A., Montes R., deFolter S., Andres-Hernandez L., 2015 The maize (*Zea mays* ssp. mays var. B73) genome encodes 33 members of the purple acid phosphatase family. Front. Plant Sci. 6: 341. 26042133 10.3389/fpls.2015.00341 PMC4436580

[bib18] Griffith M., Timonin M., Wong A., Gray G., Akhter S., 2007 *Thellungiella*: an *Arabidopsis*-related model plant adapted to cold temperatures. Plant Cell Environ. 30: 529–538. 10.1111/j.1365-3040.2007.01653.x 17407531

[bib19] Haerty W., and Ponting C., 2015 Unexpected selection to retain high GC content and splicing enhancers within exons of multiexonic lncRNA loci. RNA 21: 333–346. 10.1261/rna.047324.114 PMC4338330 25589248

[bib20] Hansen T., 1997 Stabilizing selection and the comparative analysis of adaptation. Evolution 51: 1341–1351. 10.1111/j.1558-5646.1997.tb01457.x 28568616

[bib21] Harmon L., Weir J., Brock C., Glor R., and Challenger W., 2008 Geiger: investigating evolutionary radiations. Bioinformatics 24: 129–131. 10.1093/bioinformatics/btm538 18006550

[bib22] Harrow J., Frankish A., Gonzalez J. M., Tapanari E., Diekhans M., 2012 GENCODE: the reference human genome annotation for The ENCODE Project. Genome Res. 22: 1760–1774. 10.1101/gr.135350.111 22955987 PMC3431492

[bib23] Hezroni H., Koppstein D., Schwartz M., Avrutin A., Bartel D., 2015 Principles of long noncoding RNA evolution derived from direct comparison of transcriptomes in 17 species. Cell Reports 11: 1110–1122. 10.1016/j.celrep.2015.04.023 25959816 PMC4576741

[bib24] Jackson R., Kroehling L., Khitun A., Bailis W., Jarret A., 2018 The translation of non-canonical open reading frames controls mucosal immunity. Nature 564: 434–438. 10.1038/s41586-018-0794-7 30542152 PMC6939389

[bib25] James A., Jayasena A., Zhang J., Berkowitz O., Secco D., 2017 Evidence for Ancient Origins of Bowman-Birk Inhibitors from *Selaginella moellendorffii*. Plant Cell 29: 461–473. 10.1105/tpc.16.00831 28298518 PMC5385957

[bib26] Kamilar J., and Cooper N., 2013 Phylogenetic signal in primate behaviour, ecology and life history. Philos. Trans. R. Soc. Lond. B Biol. Sci. 368: 20120341 10.1098/rstb.2012.0341 23569289 PMC3638444

[bib27] Kapranov P., Cheng J., Dike S., Nix D. A., Duttagupta R., 2007 RNA maps reveal new RNA classes and a possible function for pervasive transcription. Science 316: 1484–1488. 10.1126/science.1138341 17510325

[bib28] Kapusta A., and Feschotte C., 2014 Volatile evolution of long noncoding RNA repertoires: mechanisms and biological implications. Trends Genet. 30: 439–452. 10.1016/j.tig.2014.08.004 25218058 PMC4464757

[bib29] Katoh K., Misawa K., Kuma K., and Miyata T., 2002 MAFFT: a novel method for rapid multiple sequence alignment based on fast Fourier transform. Nucleic Acids Res. 30: 3059–3066. 10.1093/nar/gkf436 12136088 PMC135756

[bib30] Kazachkova Y., Eshel G., Pantha P., Cheeseman J., Dassanayake M., 2018 Halophytism: What Have We Learnt From *Arabidopsis thaliana* Relative Model Systems? Plant Physiol. 178: 972–988. 10.1104/pp.18.00863 30237204 PMC6236594

[bib31] Keck F., Rimet F., Bouchez A., and Franc A., 2016 phylosignal: an R package to measure, test, and explore the phylogenetic signal. Ecol. Evol. 6: 2774–2780. 10.1002/ece3.2051 27066252 PMC4799788

[bib32] Lang D., Ullrich K., Murat F., Fuchs J., Jenkins J., 2018 The *Physcomitrella patens* chromosome-scale assembly reveals moss genome structure and evolution. Plant J. 93: 515–533. 10.1111/tpj.13801 29237241

[bib33] Liu W., Cheng C., Lin Y., XuHan X., and Lai Z., 2018 Genome-wide identification and characterization of mRNAs and lncRNAs involved in cold stress in the wild banana (*Musa itinerans*). PLoS One 13: e0200002 10.1371/journal.pone.0200002 29985922 PMC6037364

[bib34] MacLeod M. J., Dedrick J., Ashton C., Sung W. W., Champigny M. J., 2015 Exposure of two *Eutrema salsugineum* (*Thellungiella salsuginea*) accessions to water deficits reveals different coping strategies in response to drought. Physiol. Plant. 155: 267–280. 10.1111/ppl.12316 25496221

[bib35] Merchant S., Prochnik S., Vallon O., Harris E., Karpowicz S., 2007 The *Chlamydomonas* genome reveals the evolution of key animal and plant functions. Science 318: 245–250. 10.1126/science.1143609 17932292 PMC2875087

[bib36] Mohammadin S., Edger P., Pires J., and Schranz M., 2015 Positionally-conserved but sequence-diverged: identification of long non-coding RNAs in the *Brassicaceae* and *Cleomaceae*. BMC Plant Biol. 15: 217 10.1186/s12870-015-0603-5 26362138 PMC4566204

[bib37] Moran P. A., 1948 The interpretation of statistical maps. J. R. Stat. Soc. B 10: 243–251.

[bib38] Nejat N., and Mantri N., 2018 Emerging roles of long non-coding RNAs in plant response to biotic and abiotic stresses. Crit. Rev. Biotechnol. 38: 93–105. 10.1080/07388551.2017.1312270 28423944

[bib39] Nelson A., Forsythe E., Devisetty U., Clausen D., Haug-Batzell A., 2016 A Genomic Analysis of Factors Driving lincRNA Diversification: Lessons from Plants. G3 (Bethesda) 6: 2881–2891. 10.1534/g3.116.030338 27440919 PMC5015945

[bib40] Niazi F., and Valadkhan S., 2012 Computational analysis of functional long noncoding RNAs reveals lack of peptide-coding capacity and parallels with 3′ UTRs. RNA 18: 825–843. 10.1261/rna.029520.111 22361292 PMC3312569

[bib41] Ouyang S., Zhu W., Hamilton J., Lin H., Campbell M., 2007 The TIGR Rice Genome Annotation Resource: improvements and new features. Nucleic Acids Res. 35: D883–D887. 10.1093/nar/gkl976 17145706 PMC1751532

[bib42] Pagel M., 1999 Inferring the historical patterns of biological evolution. Nature 401: 877–884. 10.1038/44766 10553904

[bib43] Panchy N., Wu G., Newton L., Tsai C., Chen J., 2014 Prevalence, evolution, and cis-regulation of diel transcription in *Chlamydomonas reinhardtii*. G3 (Bethesda) 4: 2461–2471. 10.1534/g3.114.015032 25354782 PMC4267941

[bib44] Paradis E., and Schliep K., 2019 ape 5.0: an environment for modern phylogenetics and evolutionary analyses in R. Bioinformatics 35:526–528. 10.1093/bioinformatics/bty633 30016406

[bib45] Pertea M., Pertea G., Antonescu C., Chang T., Mendell J., 2015 StringTie enables improved reconstruction of a transcriptome from RNA-seq reads. Nat. Biotechnol. 33: 290–295. 10.1038/nbt.3122 25690850 PMC4643835

[bib46] Revell L., Harmon L., and Collar D., 2008 Phylogenetic signal, evolutionary process, and rate. Syst. Biol. 57: 591–601. 10.1080/10635150802302427 18709597

[bib47] Rivas E., Clements J., and Eddy S., 2017 A statistical test for conserved RNA structure shows lack of evidence for structure in lncRNAs. Nat. Methods 14: 45–48. 10.1038/nmeth.4066 27819659 PMC5554622

[bib48] Saini H., Enright A., and Griffiths-Jones S., 2008 Annotation of mammalian primary microRNAs. BMC Genomics 9: 564 10.1186/1471-2164-9-564 19038026 PMC2632650

[bib49] Schnable P., Ware D., Fulton R., Stein J., Wei F., 2009 The B73 maize genome: complexity, diversity, and dynamics. Science 326: 1112–1115. 10.1126/science.1178534 19965430

[bib50] Sharma S., Bolser D., deBoer J., Sonderkaer M., Amoros W., 2013 Construction of reference chromosome-scale pseudomolecules for potato: integrating the potato genome with genetic and physical maps. G3 (Bethesda) 3: 2031–2047. 10.1534/g3.113.007153 24062527 PMC3815063

[bib51] Shuai P., Liang D., Tang S., Zhang Z., Ye C., 2014 Genome-wide identification and functional prediction of novel and drought-responsive lincRNAs in *Populus trichocarpa*. J. Exp. Bot. 65: 4975–4983. 10.1093/jxb/eru256 24948679 PMC4144774

[bib52] Simopoulos C., Weretilnyk E., and Golding G., 2018 Prediction of plant lncRNA by ensemble machine learning classifiers. BMC Genomics 19: 316 10.1186/s12864-018-4665-2 29720103 PMC5930664

[bib53] Sprenger H., Kurowsky C., Horn R., Erban A., Seddig S., 2016 The drought response of potato reference cultivars with contrasting tolerance. Plant Cell Environ. 39: 2370–2389. 10.1111/pce.12780 27341794

[bib54] Taji T., Seki M., Satou M., Sakurai T., Kobayashi M., 2004 Comparative genomics in salt tolerance between *Arabidopsis* and *Arabidopsis*-related halophyte salt cress using *Arabidopsis* microarray. Plant Physiol. 135: 1697–1709. 10.1104/pp.104.039909 15247402 PMC519083

[bib55] Tomato Genome Consortium, 2012 The tomato genome sequence provides insights into fleshy fruit evolution. Nature 485: 635–641. 10.1038/nature11119 22660326 PMC3378239

[bib56] Velasco V., Mansbridge J., Bremner S., Carruthers K., Summers P., 2016 Acclimation of the crucifer *Eutrema salsugineum* to phosphate limitation is associated with constitutively high expression of phosphate-starvation genes. Plant Cell Environ. 39: 1818–1834. 10.1111/pce.12750 27038434

[bib57] Wang D., Qu Z., Yang L., Zhang Q., Liu Z., 2017 Transposable elements (TEs) contribute to stress-related long intergenic noncoding RNAs in plants. Plant J. 90: 133–146. 10.1111/tpj.13481 28106309 PMC5514416

[bib58] Wang H. V., and Cheksnova J. A., 2017 Long Noncoding RNAs in Plants, Long Non Coding RNA Biology, edited by Rao M. R. S. Springer, Singapore. Nature 10.1007/978-981-10-5203-3_5

[bib59] Wilkins O., Hafemeister C., Plessis A., Holloway-Phillips M., Pham G., 2016 EGRINs (Environmental Gene Regulatory Influence Networks) in Rice That Function in the Response to Water Deficit, High Temperature, and Agricultural Environments. Plant Cell 28: 2365–2384. 10.1105/tpc.16.00158 27655842 PMC5134975

[bib60] Wong C., Li Y., Labbe A., Guevara D., and Nuin P., 2006 Transcriptional profiling implicates novel interactions between abiotic stress and hormonal responses in *Thellungiella*, a close relative of *Arabidopsis*. Plant Physiol. 140: 1437–1450. 10.1104/pp.105.070508 16500996 PMC1435811

[bib61] Woo H., Koo H., Kim J., Jeong H., Yang J., 2016 Programming of Plant Leaf Senescence with Temporal and Inter-Organellar Coordination of Transcriptome in *Arabidopsis*. Plant Physiol. 171: 452–467. 10.1104/pp.15.01929 26966169 PMC4854694

[bib62] Xiao L., Yang G., Zhang L., Yang X., Zhao S., 2015 The resurrection genome of *Boea hygrometrica*: A blueprint for survival of dehydration. Proc. Natl. Acad. Sci. USA 112: 5833–5837. 10.1073/pnas.1505811112 25902549 PMC4426394

[bib63] Xu Q., Song Z., Zhu C., Tao C., Kang L., 2017 Systematic comparison of lncRNAs with protein coding mRNAs in population expression and their response to environmental change. BMC Plant Biol. 17: 42 10.1186/s12870-017-0984-8 28193161 PMC5307861

[bib64] Yang R., Jarvis D., Chen H., Beilstein M., Grimwood J., 2013 The Reference Genome of the Halophytic Plant *Eutrema salsugineum*. Front. Plant Sci. 4: 46 10.3389/fpls.2013.00046 23518688 PMC3604812

[bib65] Yin J., Gosney M. J., Dilkes B. P., and Mickelbart M. V., 2018 Dark period transcriptomic and metabolic profiling of two diverse *Eutrema salsugineum* accessions. Plant Direct 2: e00032 10.1002/pld3.32 31245703 PMC6508522

[bib66] Zhao X., Li J., Lian B., Gu H., Li Y., 2018 Global identification of Arabidopsis lncRNAs reveals the regulation of MAF4 by a natural antisense RNA. Nat. Commun. 9: 5056 10.1038/s41467-018-07500-7 30498193 PMC6265284

[bib67] Zhu Y., Wang B., Phillips J., Zhang Z., Du H., 2015 Global Transcriptome Analysis Reveals Acclimation-Primed Processes Involved in the Acquisition of Desiccation Tolerance in *Boea hygrometrica*. Plant Cell Physiol. 56: 1429–1441. 10.1093/pcp/pcv059 25907569

